# Distinct spatio-temporal and spectral brain patterns for different thermal stimuli perception

**DOI:** 10.1038/s41598-022-04831-w

**Published:** 2022-01-18

**Authors:** Zied Tayeb, Andrei Dragomir, Jin Ho Lee, Nida Itrat Abbasi, Emmanuel Dean, Aishwarya Bandla, Rohit Bose, Raghav Sundar, Anastasios Bezerianos, Nitish V. Thakor, Gordon Cheng

**Affiliations:** 1 Institute for Cognitive Systems, Technical University of Munich, Arcisstraße 21, 80333 Munich, Germany; 2 The N.1 Institute for Health, National University of Singapore, 28 Medical Dr. 05-COR, Singapore, 117456 Singapore; 3 Department of Biomedical Engineering, University of Houston, 3517 Cullen Blvd, Houston, TX 77204 USA; 4 Chalmers University of Technology, 412 96 Gothenburg, Sweden; 5 Department of Bioengineering, University of Pittsburgh, 3700 O’Hara Street, Pittsburgh, PA 15261 USA; 6 Department of Haematology-Oncology, National University Cancer Institute, National University Hospital, 5 Lower Kent Ridge Rd, Singapore, 119074 Singapore; 7 Hellenic Institute of Transport (HIT), Centre for Research and Technology (CERTH), Thessaloniki, Greece; 8 Department of Biomedical Engineering, Johns Hopkins School of Medicine, 720 Rutland Ave, Baltimore, MD 21205 USA; 9 Department of Biomedical Engineering, National University of Singapore, Engineering Drive 3, #04-08, Singapore, 117583 Singapore

**Keywords:** Engineering, Biomedical engineering

## Abstract

Understanding the human brain’s perception of different thermal sensations has sparked the interest of many neuroscientists. The identification of distinct brain patterns when processing thermal stimuli has several clinical applications, such as phantom-limb pain prediction, as well as increasing the sense of embodiment when interacting with neurorehabilitation devices. Notwithstanding the remarkable number of studies that have touched upon this research topic, understanding how the human brain processes different thermal stimuli has remained elusive. More importantly, very intense thermal stimuli perception dynamics, their related cortical activations, as well as their decoding using effective features are still not fully understood. In this study, using electroencephalography (EEG) recorded from three healthy human subjects, we identified spatial, temporal, and spectral patterns of brain responses to different thermal stimulations ranging from extremely cold and hot stimuli (very intense), moderately cold and hot stimuli (intense), to a warm stimulus (innocuous). Our results show that very intense thermal stimuli elicit a decrease in alpha power compared to intense and innocuous stimulations. Spatio-temporal analysis reveals that in the first 400 ms post-stimulus, brain activity increases in the prefrontal and central brain areas for very intense stimulations, whereas for intense stimulation, high activity of the parietal area was observed post-500 ms. Based on these identified EEG patterns, we successfully classified the different thermal stimulations with an average test accuracy of 84% across all subjects. En route to understanding the underlying cortical activity, we source localized the EEG signal for each of the five thermal stimuli conditions. Our findings reveal that very intense stimuli were anticipated and induced early activation (before 400 ms) of the anterior cingulate cortex (ACC). Moreover, activation of the pre-frontal cortex, somatosensory, central, and parietal areas, was observed in the first 400 ms post-stimulation for very intense conditions and starting 500 ms post-stimuli for intense conditions. Overall, despite the small sample size, this work presents novel findings and a first comprehensive approach to explore, analyze, and classify EEG-brain activity changes evoked by five different thermal stimuli, which could lead to a better understanding of thermal stimuli processing in the brain and could, therefore, pave the way for developing a real-time withdrawal reaction system when interacting with prosthetic limbs. We underpin this last point by benchmarking our EEG results with a demonstration of a real-time withdrawal reaction of a robotic prosthesis using a human-like artificial skin.

## Introduction

Over the last decades, the quantification of noxious sensations using neuroimaging techniques has been widely studied and investigated^1–3^. Despite the significant number of studies in this context^3–6^, understanding nociceptive processing in the human brain is still very challenging^3,6,7^. This is due to the complex mechanisms involved in processing very intense stimuli. Similarly, such stimuli perception and processing are likely to be harbored in different regions in the brain^6,8^. En route to analyzing and unraveling the underlying processes within the human body that can lead to the unpleasant sensation perception, understanding intense thermal sensation, in particular, has recently gained momentum^9–11^. Neuroscientists have been relying on various neuroimaging techniques, including functional magnetic resonance imaging (fMRI)^12^, Positron emission tomography (PET)^13^, and invasive recordings^14^, to investigate exogenous pain processing. Kwan et al.^12^ investigated in their study using fMRI the ACC’s role in sensory, motor, and cognitive functions when perceiving four thermal stimuli categorized into innocuous (cool, warm) and noxious conditions (cold, hot). However, the correlation between EEG evoked changes and different nociceptive thermal stimuli have been minimally studied^11,15^. Overall, EEG offers a better temporal resolution and has not been extensively studied in this context. In the study by Mulders et al.^11^, noxious heat and innocuous cool stimuli were applied to the forearm of healthy subjects. Particularly, they focused on both heat- and cold-evoked steady-state response. They reported and concluded that both stimuli were associated with a periodic EEG response at 0.2 Hz and its harmonics, and the EEG response related to cool stimulation had a much lower magnitude and shorter latency compared to the EEG response elicited by warm stimulation. In tonic pain stimulation generated by thermal stimuli^16^, Backonja et al. analyzed EEG power spectra changes and reported an increase in alpha [8–12 Hz] and beta [13–25 Hz] power^16^. Along the same lines, Ploner et al.^17^ investigated the different early temporal event-related waves, as well as frequency responses in the Gamma band [25–140 Hz]. Interestingly, another study^16^ reported a significant difference between painful and non-painful stimulations, which was detected in both parietal and frontal areas. Thus, this could be explained by motor withdrawal responses. In the same study^16^, an EEG frequency domain analysis was performed and alpha-band’s event-related desynchronization (ERD) was detected when performing hand immersion into cool and extremely cold water (noxious). This initial decrease of alpha-band was followed by an increase in bilateral frontal and posterior regions. Similarly, in Chen et al.^18^, a pronounced decrease of alpha magnitude in the central areas was detected in EEG recordings during an experimental ice-cube cold pressor test. In a different study, Hu et al.^19^ studied the relationship between EEG brain responses and $A\delta$- and C-fibre skin nociceptors. By investigating $A\delta$- and C-fiber laser evoked potentials, they could provide quantitative analysis about latency responses of these fibers and the underlying perception-response functions depending on the stimulus as well as the exact stimulated area. Along the same lines, Lv et al.^20^ used EEG signals to study the effects of stimulus mode and ambient temperature on cerebral responses during local thermal stimulation on hand. Interestingly, the authors reported an alternation of EEG recordings between thermally stimulated periods and the baseline in all the four EEG frequency bands. The study concluded that neural responses in different EEG frequency bands were sensitive to different factors when thermally stimulating subjects’ hands. Furthermore, the study by Wang et al.^21^ studied changes in EEG rhythms, after laser-induced heat thermal stimulation of 33–41 $^{\circ }$C. The authors reported a decrease in EEG power topographic patterns as a result of the laser-induced heat stimulation. In another study by Breton et al.^22^, they focused on power modulation of different oscillatory components and its sensitivity to thermal comfort variations using EEG recordings. The authors showed the direct modulation of EEG in different frequency bands and the thermal conditions, as well as the direct correlation with thermal comfort modulations. Power in the theta band increased in the heat stress phase, compared to the pre-stimulation and the recovery phases. In this study, we fill the gap by identifying spatial, spectral, and temporal brain patterns that can be used to distinguish a wider spectrum of thermal stimulations (five thermal stimuli) ranging from very cold to very hot stimuli. Additionally, we developed an offline system for classifying the different conditions from recorded EEG responses.

**Figure 1:**
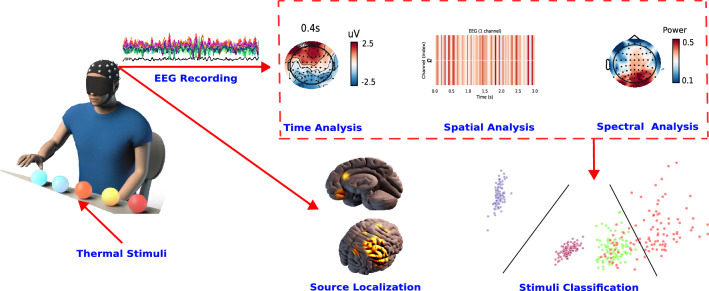
Overview of the thermal sensations processing and analysis system.

Unlike previous studies, EEG is also used to quantify brain changes for each of the five thermal stimulations. For that, brain activity is investigated at the source level, reporting new findings on the brain anticipatory activity of such stimuli and their connections to motor withdrawal preparation. To the best of our knowledge, despite the limited number of subjects, this work is the first study to provide such a comprehensive analysis and take advantage of the EEG’s temporal resolution to investigate brain responses to a wide range of thermal stimuli, as well as to accurately (84%) classify them based on the identified brain patterns. The ultimate goal of this study is to use these patterns to develop a real-time withdrawal reaction system when interacting with neurorehabilitation devices^23^. The benefits of using EEG signals instead of using the robot’s internal reaction would be to reduce the reaction latency to a couple of milliseconds (thanks to the high EEG temporal resolution), increase the sense of ownership, and enhance the intuitiveness of the prosthesis’ control. More importantly, the identification of such brain patterns has various clinical applications, including the prediction of phantom-limb pain and boosting the sense of agency for amputees when controlling their prostheses. Similarly, this could provide a tool for objective acute and chronic pain assessment for impaired patients, as well as monitor the effect of analgesic drugs. An overview of the proposed system is illustrated in Fig. 1.

## Results

### Thermal stimuli elicit a decrease in EEG alpha power

Five thermal stimuli (very cold, cold, very hot, hot, and warm) were delivered to three healthy subjects’ right hands using a customized thermal stimulator. Based on the collected behavioral data, these five conditions were clustered into three categories: very intense conditions denoted by NOX (very cold and very hot stimuli), moderately intense conditions denoted by MOD (cold and hot stimuli), and an innocuous stimulus denoted by INNO (warm stimulus). Throughout the experiment, a thermal sensation scale was used. The NOX stimulations were reported by all the three participants as uncomfortable, the MOD stimulations were reported as slightly less noticeable/intense compared to the NOX stimulation, and the INNO was reported as pleasant. The $mean \pm SD$ subjective responsivity of very cold, very hot, cold, hot, and warm was $6.6 \pm 0.48$ , $5.9 \pm 0.83$, $4.5 \pm 0.5$, $3.9 \pm 0.3$, $2.6 \pm 0.48$, respectively, on a thermal sensation scale from 0 to 9, with 9 being an intolerable noxious sensation. EEG signal was used to quantify brain activity during each thermal stimulation and to understand oscillatory neural responses en route to identifying specific brain patterns for very intense thermal stimuli processing in the brain. For that, averaged EEG alpha power (8–12 Hz) across all trials was analyzed. Our results reveal a suppression of alpha oscillatory activities characterized by a significant and consistent decrease in the alpha frequency band power [P $<0.001$, Kruskal–Wallis test was combined with the post-hoc (Tukey HSD tests)].

**Figure 2:**
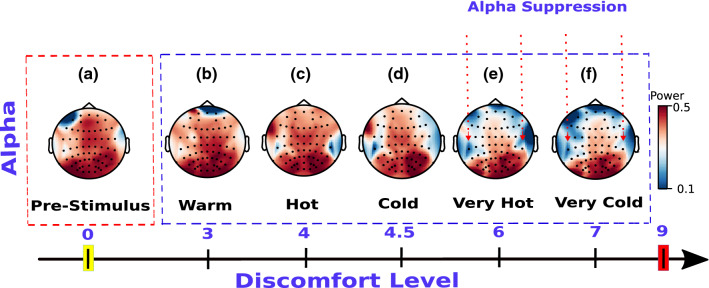
EEG alpha power topographic maps. (**a**), (**b**), (**c**), (**d**), (**e**), and (**f**) represent topographic scalp maps of the EEG alpha power for pre-stimulus, INNO (warm), MOD (hot and cold), and NOX (very hot and very cold) stimuli, respectively, using the average of all trials for each condition and across all three subjects. Suppression of alpha oscillatory activities was found in MOD and NOX conditions (**c**,**d**,**e**,**f**), whereas no decrease in alpha power was detected for the pre-stimulus and INNO conditions (**a**,**b**). The discomfort level represents the average of all subjects’ rating of each condition on a thermal sensation scale from 0 to 9.

It should be noted that this power decrease is detected ipsilaterally and contralaterally but is dominantly observed at contralateral-central electrodes (as presented in Fig. 2). Our analysis reveals a weak correlation between alpha power and the stimulation condition’s discomfort level (Pearson’s correlation r = − 0.386) at contralateral-central electrodes (left side of the brain) (P $<0.05$). Thus, the more intense the condition is, the higher and more visible the decrease is (as shown in Fig. 2).

### Spatial-temporal patterns for thermal stimuli processing in the brain

**Figure 3:**
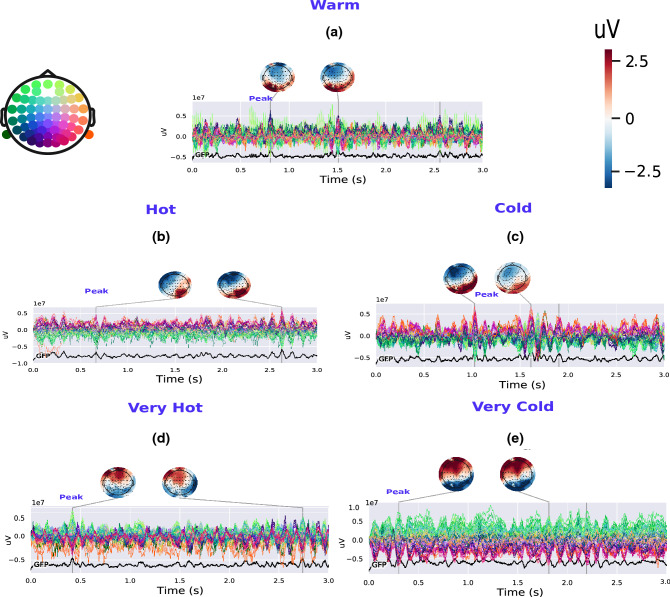
Spatial-temporal EEG features for all five thermal conditions. (**a**), (**b**), (**c**), (**d**), (**e**), and (**f**) represent topographic scalp maps of the EEG amplitude response for warm, hot, cold, very hot, and very cold stimuli, respectively, using the average of all trials for each condition and across all subjects. All topographic maps are plotted for the first three-second post-stimulation time window (average across each condition’s trials). Peaks are detected at 0.3 s, 0.45 s, 0.65 s, 0.95 s, and 0.8 s, for very cold, very hot, hot, cold, and warm, respectively. The get_peak algorithm in the MNE software^24^ is used to compute and detect the amplitude of the maximum EEG response (local maxima) for NOX as well as the location (EEG channel) and latency of the detected peak amplitude. Time courses of NOX stimulation, identified and found using the get_peak algorithm were used thereafter for analysis and benchmarking with the other two conditions. The global field power (GPF) is plotted for each stimulation condition.

The aforementioned alpha power topography analysis was extended by aiming to identify distinct brain patterns distinguishing between the five thermal stimuli and to investigate spatial and temporal brain responses for each stimulus. For both NOX stimulations, we find out, as shown in Fig. 3d,e that the highest brain activation is detected at the pre-frontal cortex, contralateral somatosensory area, and the central cortex compared to the other three thermal stimulations. For MOD conditions (cold and hot stimuli), the highest brain response is observed in the parietal area, mainly on the right parietal electrodes, with a central and frontal deactivation compared to NOX conditions (Fig. 3b,c) [p $<0.001$, Kruskal–Wallis test was combined with the post-hoc (using Tukey HSD test) for multiple group comparison]. No significant activation is detected for the warm condition, which was reported as a pleasant sensation.

**Figure 4:**
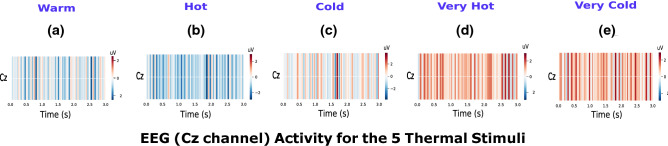
Cz activity for all five thermal conditions. (**a**), (**b**), (**c**), (**d**), and (**e**) represent EEG activity in Cz for warm, hot, cold, very hot, and very cold stimulus, respectively. The plot shows that the lowest Cz activity is detected for INNO and the highest for the NOX stimulation condition.

Additionally, as depicted in Fig. 4d,e, the highest activation for NOX conditions is localized at the vertex electrode in the middle of the scalp (Cz) when compared to MOD and warm stimuli (Fig. 4a–c) with relatively high activation also being detected in C3 and C5 electrodes. Thus, Cz activity increases with the condition’s discomfort level as shown in Fig. 4 with the highest detected Cz activity is for the very cold stimulus. Our results reveal that the highest activation for NOX conditions was detected in the interval of the first 450 ms post-stimulation ([0– 450 ms]) as shown in Fig. 3d,e, whereas the highest activation for the other two groups was observed in the interval of [500–1000 ms] post-stimulation as presented in Fig. 3b,c. Based on our findings, we postulate that the NOX stimulations lead to high and continuous activation of the central lobe in the brain, which is responsible for motor movement planning. We wish to mention that no specific spatial-temporal patterns were found in the pre-stimulus phase whose results are presented in the Supplementary Fig. S2.

### Classification of the five different thermal stimulation conditions

After investigating spectral, and spatio-temporal patterns for the five different stimuli, we sought to classify them.

**Figure 5:**
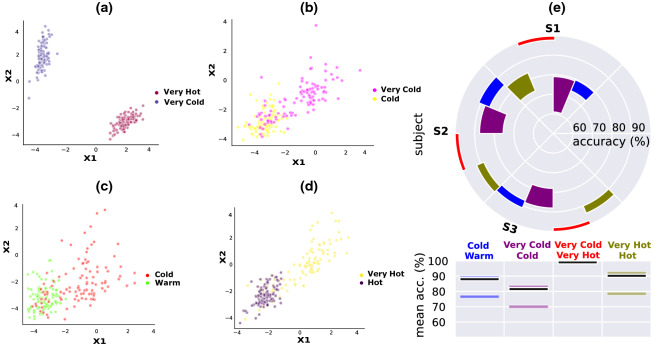
2D feature space after performing PCA highlights a clear separation between the different sub-classes. $\times$ 1 and $\times$ 2 represent the first two components after performing PCA. (**a**), (**b**), (**c**), and (**d**) represent the feature space when classifying (very hot, very cold), (very cold, cold), (cold, warm), and (very hot, hot), respectively. (**e**) shows the validation accuracy for the three subjects (S1, S2, S3) and for the classification tasks using a polar bar plot. This circular plot shows the accuracy range $mean \pm SD$ achieved by the three different subjects. The three horizontal bars represent the min, median (black bar), and max accuracy values.

Unlike previous studies^15,25^, where the focus was either on classifying hot and cold stimuli or distinguishing between pleasant and noxious stimuli, the novelty of this study resides in the ability to distinguish between five different temperature levels. We wish to mention that the focus of this study was not to do a multi-class classification task but rather to distinguish between specific pairs of thermal stimuli, which are difficult to classify and have not been addressed before in the literature. In the present study, we are able to achieve a mean balanced accuracy (bACC) of ${86.89}{\%} \pm {3}{\%}$, ${78.16}{\%} \pm {6.66}{\%}$, ${84.53}{\%} \pm {3}{\%}$, ${70.5}{\%} \pm {3.1}{\%}$, and ${100}{\%}$, when classifying very hot and hot, very cold and cold, warm and cold, warm and hot, and very hot and very cold stimuli, respectively, yielding an average validation accuracy of 87.4% within all classified conditions and across the three subjects. Classification results are summarized in Fig. 5. A mean bACC of 84% is obtained in the test phase within all sub-classes and across all the three subjects. For that, data were split into 80% and 20% where stratified ten-fold cross-validation was performed on 80% of the data (validation phase) and the saved model was used to predict correct labels on the remaining 20% of the trials in the test phase. Principal component analysis (PCA) was performed on the extracted features, which resulted in a clear separation between the different classes as shown in Fig. 5. Figure 5 shows that very hot and very cold are easily classified, whereas very cold and cold are hardly distinguishable. It should also be noted that warm and hot are very close to each other, and hence we did not obtain a high accuracy (70.5%) when differentiating between the two conditions using EEG signals. By analyzing each individual subject’s performance, we observe a high variability between the three subjects. The third subject (S3) clearly outperforms the others (Fig. 5e) with an average accuracy of 88.5% for all the four classification tasks, whereas S2 and S1 reach an average validation accuracy of 83.4% and 80.15%, respectively.

### Very intense stimuli induce high and early activation of the ACC

To extend our findings and analysis, we studied brain activity at the source level for the five stimulation conditions, grouped into the three classes: NOX, MOD, and INNO.

**Figure 6:**
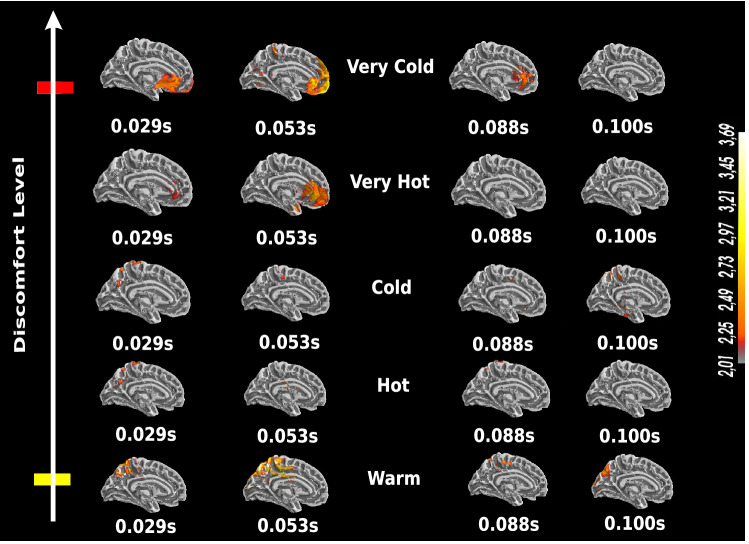
ACC-EEG activity analysis for the NOX stimuli, as well as for INNO stimulus in the first 100 ms (medial view). The dynamic statistical parametric maps (dSPM) technique^32^ is used to compute the reconstructed sources. The used scale represents the EEG amplitude activity in uV. Continuous and high EEG activity in the ACC starting 20 ms post-stimulus for NOX stimuli which is only detected for the first 100 ms. MOD and INNO conditions elicit an activation of the parietal lobe.

Localized sources using EEG signals were used to gain insights into thermal stimulus processing in the brain and the different activated regions involved in the perception of each of the three groups. All five conditions were investigated as shown in Fig. 6. For that purpose, we reconstructed EEG source dynamics using distributed source modeling^26,27^ based on realistic head models^28,29^. As illustrated in Fig. 6, only NOX conditions show activation of the mid-frontal cortex, including the ACC. Interestingly, this activation does not persist over time and is only detectable in the 100 ms post-stimulus. Particularly, very cold stimulus elicits an early response of the ACC at 29 ms and is followed by stronger activation at 53 ms post-stimulus, which diminishes over time. Similarly, as shown in Fig. 6, the very hot stimulus, which was rated less intense than the very cold, elicits activation of the ACC at around 29 ms post-stimulus and solely persisted for the first 50 ms. For the remaining conditions, no ACC activity is detected and a parietal activity is clearly visible in the first 100 ms post stimulation. Although the latencies of activations are not compatible with conduction velocities of nociceptive and thermal nerve fibers, we interpret this as an anticipatory brain behavior, given that subjects knew in advance (with the sound beep that was played 5 s prior to the actual stimulation) that they might be exposed to a very intense stimulus. Overall, the increase in ACC activation under the extreme conditions presents direct evidence of ACC’s role in activating the brain’s autonomic functions and attention circuitry, as well as an important role in external sensory stimuli perception^30,31^. Thus, this is in accordance with previous studies using different types of stimuli. As the highest and earliest ACC activation is found for the most NOX condition, the time snippets for it are used as a reference when analyzing the other conditions.

### Very intense stimuli elicit high activation of the pre-frontal, frontal and central cortex, somatosensory areas, as well as the parietal lobe

**Figure 7:**
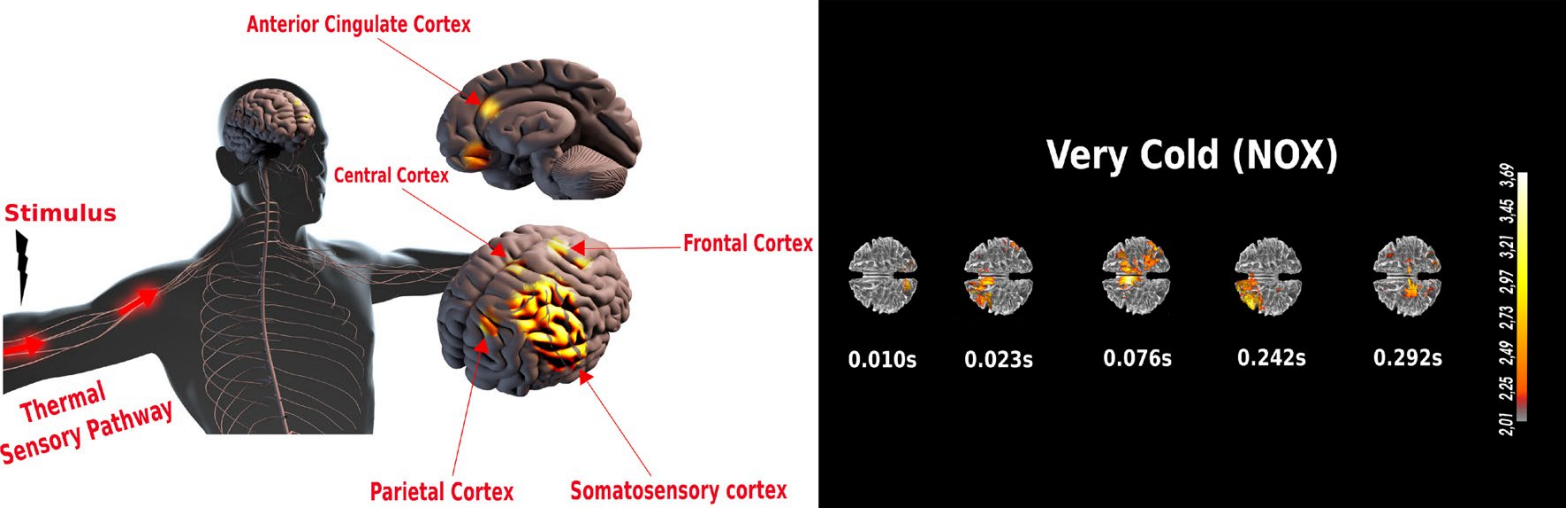
Main activated region for the very intense stimuli after performing EEG source localization. ACC, central cortex, pre-frontal and parietal areas are the main activated brain regions for NOX conditions (left). NOX condition localized sources with brain time responses in the first 300 ms window of post stimulation.

Next, we attempted to characterize changes in cortical activity during perception of the five different thermal stimuli. For our extended source localization analysis, we solely focused on the most NOX condition (very cold) en route to understanding the brain circuitry involved in very intense thermal sensation processing. Overall, we observed that brain activity associated with NOX condition activates more brain regions when compared to to MOD and INNO stimulations (Fig. 6). Our results reveal that NOX sensations elicit high activation of the pre-frontal, frontal and central cortex, somatosensory areas, as well as the right parietal cortex. Similarly, we observe that, for NOX conditions, contralateral activity is significantly higher than the ipsilateral one, which is aligned with the literature^16^. These activated regions seem to form a complex interconnected circuitry for very intense stimulus perception and processing as was reported in the literature^16^. Figure 7 (left) summarizes the main activated brain regions for NOX conditions using EEG localized sources, whereas the right side of Fig. 7 highlights the time response of those activated regions. Overall, as shown in Fig. 7, the most NOX stimulus induces activation of the parietal areas, which is followed by an activation of both frontal (23 and 242 ms) and central area (76 and 292 ms). Despite the incompatibility with conduction velocities of nociceptive and thermal nerve fibers, these early activations could be explained by an anticipatory brain activity.

### The robotic application: a reflex system in robotic hands that can mimic the natural human hand when reacting to the same five thermal stimuli

**Figure 8:**
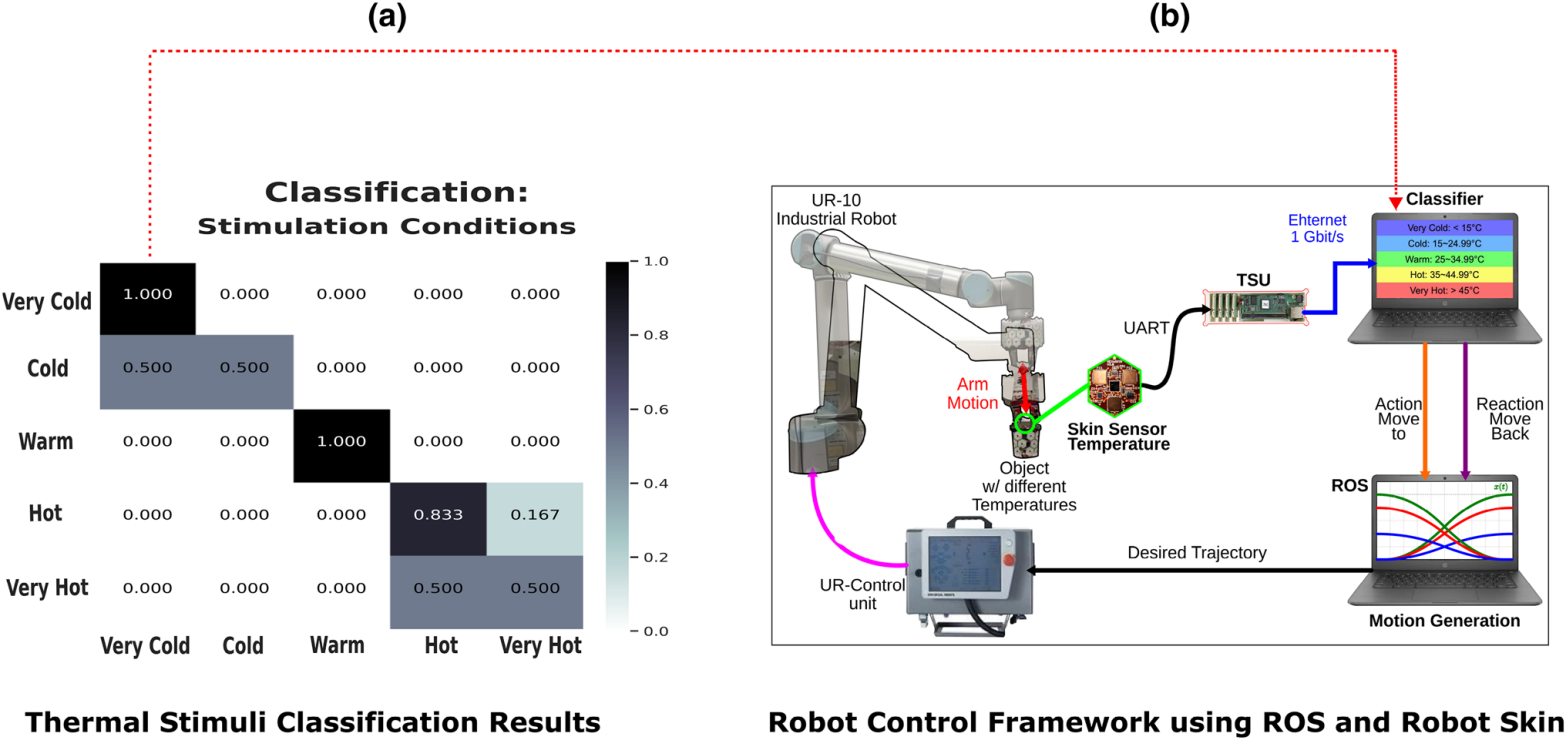
Overview of the real-time robotic experiment, showing a robot arm that can feel, distinguish, and react to the same five thermal stimuli. (**a**) shows the confusion matrix for the real-time experiment when classifying the five thermal stimuli. (**b**) shows the whole work flow, the sensing part using the robot skin, stimuli classification, and the robot control framework using the robot operating system (ROS).

The ultimate goal of our study is to develop a real-time withdrawal reaction when amputees interact with their prosthetic limbs, which can first protect these devices from damaging stimuli, but particularly, increase the sense of agency^33^, the intuitiveness of control^34,35^, and more importantly, reduce the withdrawal reaction time using the effective identified EEG neuronal features. As a first step, we benchmarked our findings and compared the effectiveness of using the identified EEG patterns when interacting with prostheses with the device’s internal reaction without the proposed human-in-the-loop approach. By investigating this other side of the equation, we developed a withdrawal reaction system in a UR-10 robot arm alongside an attached prosthetic hand with no human-in-the-loop. The robotic hand equipped with human-like skin, so-called robot skin^36^, can distinguish between the same above-mentioned five thermal stimuli with a validation accuracy of 92.6% and real-time test accuracy of 84%. It should be noted that when running this experiment for a long time, very cold and very hot were confused with hot and cold, as objects’ temperature changed when performing long-time recordings, which could explain the performance (accuracy) decrease in the real-time setup as shown in Fig. 8b. Overall, based on the object’s temperature and the classified type of thermal stimulus (INNO, MOD, and NOX), the robot arm withdraws at different speeds: delayed (very slow) for INNO, slow for MOD, and fast for NOX stimuli. The system and the main results are illustrated in Fig. 8. A video of a successful live demo is available in the Supplementary Information section. As shown in the demo video in the Supplementary Information, the withdrawal reaction time could be reduced, when using EEG neuronal patterns, from 3 s to 750 ms, and hence allowing for an immediate reaction system in prostheses. Ultimately, using real-time EEG patterns, robotic prostheses will mimic the actual human hand while substituting the role played by the reflexive motor neurons in the spinal cord.

## Discussion

In this study, temporal, spatial, and spectral EEG changes for five different thermal stimuli were characterized using brain signals collected from three healthy subjects. EEG was used to quantify brain changes and to develop distinct patterns for five different thermal stimuli because of its good compromise between the level of invasiveness and high temporal resolution^37^. Overall, the main findings of this work concur with previous reports of brain-related activation for noxious-evoked stimuli. To the best of our knowledge, this study is the first to propose a comprehensive analysis of brain activity using non-invasive EEG recordings for five thermal stimuli ranging from extremely cold to extremely hot sensations. Similarly, this study presents new findings on how five thermal stimulations are processed in the brain and uses the identified patterns to distinguish between the different sensation levels, showing the potential of such an approach for real-time human–robot interaction.

### Neural patterns of thermal stimuli-evoked activity

As shown in Fig. 2, EEG-alpha power was found to be associated with stimulus intensities. Overall, a pronounced decrease of alpha power in the central areas was identified for NOX conditions. Although this decrease was detected contralaterally and ipsilaterally, it was significantly higher on the contralateral side of the stimulation. We postulate that this significant power difference is explained by an ERD^38,39^, which is usually evoked by phasic endogenous or exogenous stimulation, frequently applied in visual, auditory, and movement experimental paradigms^40^. As this decrease was mainly detected in central electrodes, overlapping with somatosensory regions, we hypothesize that for NOX conditions, subjects had the intention to move their limbs away from the very intense stimulus, which potentially resulted in a motor imagery movement^41,42^, and led to a clear ERD pattern similar to the one widely used in brain–computer interfaces. Overall, our results concur with most previous studies investigating the relationship between EEG-alpha power and noxious stimuli^16,43^, as well as further extend upon these studies by analyzing five distinct thermal conditions. In another study^16^ where only two conditions (cold and cool water) were investigated, Backonja et al. reported an initial decrease in alpha power in the first minute post-stimulus, which was thereafter followed by an alpha power increase in bilateral frontal and posterior electrodes. Similarly, they reported that cool (warm) stimulation produced less alpha power change than the cold condition. Along the same lines, the pronounced decrease of alpha amplitude in the central areas was also detected in Ref.^18^, where subjects participated in an ice-cube cold pressor experiment. It should be noted, however, that in some previous studies noxious stimulations were instead associated with an increase in alpha activities^44^. This discrepancy is likely to be explained by the chosen stimulation type or subject’s age difference, as reported in the study^44^, where transcutaneous electrical nerve stimulation (TENS) was used to elicit unpleasant sensations and was tested for both young and elderly people. When analyzing the other EEG frequency bands, we also observed power decrease (as in alpha power) in the theta and beta frequency bands, as was also reported in previous related studies, whereas an overall increase in the power of gamma-band was observed^5,45–47^. As no statistical significance was found when analyzing theta, beta, and gamma frequency bands, their corresponding results are presented in the supplementary materials. As depicted in Fig. 3, our results reveal that NOX conditions generated high activity around Cz central electrode compared to MOD and INNO conditions. Additionally, as shown in Fig. 3, NOX stimuli generated higher brain activity in the first 450 ms post-stimulus, whereas MOD and warm conditions showed higher brain activity starting 500 ms post-stimulus. We hypothesize that only the NOX conditions trigger a withdrawal reaction, reflecting the subjects’ intention to escape the potential source of very intense thermal sensation. The latter heavily involves central brain areas, and hence explains the higher activation of Cz electrode for NOX stimuli compared to the other stimulation conditions. This result is in accordance with previous studies where high activation Cz was also reported when using different NOX stimuli with amputees^48^ and infants^49^.

### Neuronal responses to varying thermal stimulus tolerability: relation with $A\delta$-skin nociceptors

Even though the interpretation of the exact mechanisms responsible for the detected latency difference when responding to different tolerable and intolerable thermal sensations remains unclear, we postulate that there seems to be a strong correlation with the $A\delta$-skin nociceptors’ time responses. Noticeably, the brain time response for NOX stimuli (as shown in Fig. 3) matches the response time of $A\delta$, as reported in this previous study^19^. $A\delta$-fibres, which are ‘fast’ myelinated nociceptive nerve fibres that respond to noxious thermal stimuli by carrying rapid nervous messages from the skin to the brain^50^ and play an important role in the ascending pain-control pathway^51^, were shown in Hu et al.^19^ to have a strong brain response in the first 400 ms after noxious stimulation. Thus, this could present evidence of their involvement in processing NOX conditions and explain the observed latency when processing the two NOX stimuli.

### ACC’s strong and early activation reveals its role in thermal sensation anticipation and focal attention

The role of ACC in thermal stimuli processing and their related cognitive functions has been widely investigated in the literature^12,52,53^. Overall, its role is believed to be associated with stimulus perception, as well as prediction and avoidance of very intense stimuli, with a particularly significant response when processing heat stimuli^12,54,55^. In the present study, we observe strong and early activation of the ACC for NOX stimuli in the first 100 ms post-stimulation (Fig. 6). Noticeably, this activation does not seem to persist after 100 ms (as shown in Supplementary Fig. 3 in the Supplementary Information). Unlike NOX stimuli, this early high ACC activation was not detected when processing the other thermal conditions. Our results reveal the important role of the ACC in the central processing of thermal stimuli^56,57^. It is worth noting that during the experiment a warning beep was used before each trial (with a fixed time), and hence the subjects knew in advance that the stimulation would happen in a few seconds following the beep. This could explain the anticipatory pattern, as the identified latencies of brain responses are not compatible with thermal fibers conductivity. Additionally, recordings were performed in two different sessions, when the subjects were exposed to one of the two NOX stimuli (either very hot of very cold), which were randomized with the MOD and INNO stimuli. As a result, we hypothesize that the subjects had expectations preceding the very intense thermal stimuli and this triggered activation of certain brain regions even before the stimulation’s occurrence, so-called “anticipation”. This seems to provide an explanation to this early ACC activation (29 ms) shown in Fig. 6, further demonstrating its role in perceptual-anticipation of NOX stimuli, and could provide an evidence that this stimulus anticipation triggered the salience network^58^. Such network pre-activates particular brain regions^59^ whose the ACC is one of the central nodes. Our findings are also in accordance with the literature, where other studies showed, using invasive neuroimaging techniques, an ACC activation 2600 ms prior to the stimulus^59^. Interestingly, as our results reveal that this ACC activation was generally associated with high fronto-central activity, this could be further explained by a fear-induced activity that the subjects had during the experiment. Although fear processing seems to be harbored in different brain regions, it was shown in a previous study^8^ that a fear-induced pattern was associated with a fronto-central activation stemming from the ACC. Similarly, as this activation persists for the 100 ms post-stimulus and solely for NOX conditions, this highlights its role not only in anticipation but also in the second phase of sensory processing and focal attention to NOX stimuli. Hence, this clear ACC activity (at 53 and 88 ms) shown in Fig. 6 could be linked to the subjects’ inhibitory intention and withdrawal behavior when being exposed to very intense thermal conditions^60^. One intuitive reason why this activation was only present for NOX conditions is that the other stimuli (INNO and MOD) did not present any potential risk and were not considered by the subjects as harmful or damaging stimuli. Additionally, as this activation was only detected for NOX conditions, we postulate that this could be explained by an internal brain model that is anticipating these stimuli and only reacting to the most intense ones. Overall, using a high-temporal resolution neuroimaging technique, our results solidify and extend upon previous findings on the essential role of the ACC as an early warning system to alert humans about potential risks, and could further explain its involvement in autonomic functions, such as body preparation to process external thermal sensations.

### Interpretation of central role of prefrontal, parietal, central, and somatosensory areas in thermal stimuli processing

In this study, we report strong brain activation of pre-frontal, frontal, somatosensory, and central areas for NOX conditions [p $<0.0001$], whereas high activation of the right parietal cortex was found for MOD and INNO conditions [p $<0.001$] as was illustrated in Figs. 6 and 3. Overall, the involvement of these cortical areas in very intense thermal sensation perception has been previously confirmed in different neurophysiological studies^16^. Particularly, Pardo et al.^2^ showed, using PET techniques, the specific role of the right posterior parietal cortex in focal attention and processing of sensory input, regardless of whether it is noxious or not. It is also proven that the parietal area is part of a larger network of sensory stimuli processing, which involves attentional allocation, orientation and preparedness for withdrawal reactions. This could provide an explanation of the observed right posterior parietal activity when processing MOD and INNO stimuli. Interestingly, this seems to involve more regions when perceiving NOX sensory stimuli, mainly bilateral prefrontal, medial frontal, pre-motor cortex and the ACC, forming a complex attention circuitry^2^. The activation of the frontal area reflects “vigilance”^13^, focal attention, and action planning^61^. Similarly, it could be also linked to its connection to the basal ganglia which has different roles in noxious stimuli processing, including sensory discrimination, information gating, and warning input to different motor areas^62,63^. We postulate that this could reveal the subjects’ intention to either move their arms when being exposed to very intense thermal stimuli (a motor imagery function) or an internal suppression and inhibition of these NOX sensations, given that they were strictly instructed not to move their limbs throughout the recording sessions. As the frontal area is heavily connected to the ACC, their observed simultaneous co-activation for very cold and very hot stimuli could be further interpreted as a stimulus evoked fear avoidance which may lead to motor initiation or suppression, and hence activate the pre-motor cortex.

### Very intense thermal sensation predictivity for engineering and clinical applications

In our study, the five thermal stimuli ranging from extremely cold to extremely hot were recognized using EEG signals with an average validation accuracy of 84% across the three subjects. To the best of our knowledge, this high classification accuracy of five distinct thermal stimuli, using non-invasive brain recordings, has not been reported before in the literature and presents clear evidence of the uniqueness and novelty of this study. Aside from real-time human–robot interaction applications, such a classification system could be used to predict unpleasant sensations even before they occur, since our ACC analysis has shown that a clear anticipation pattern in the first 100 ms post noxious stimuli could be detected. Thus, this can be used for clinical applications, such as phantom-limb pain mitigation^64^ and chronic pain treatment^65^. As was shown in our results, inter-subject performance differences were clearly identified. We postulate that this could be explained by the fact that people tend to perceive, behave and react differently to NOX, MOD, and INNO stimulations, depending on many external uncontrolled factors, such as age, country of origin, and gender. Hence, further improvement of the classification system and its robustness should be performed.

### Towards a real-time withdrawal reaction system in robotic prostheses

The scope of this study goes beyond understanding the underlying mechanisms of thermal stimuli processing and perception in the brain, as well as the identification of brain features. Thus, our ultimate goal is to develop a real-time withdrawal system in embodied prostheses^48^ en route to increasing the sense of embodiment and agency^66^. Here, we propose a new and low-latency system of detecting thermal sensation in the brain based on EEG signals. Additionally, we underpin the potential of this system from a robotic perspective, by demonstrating a robotic prosthesis that can sense, distinguish, and react to the same five thermal stimuli. The purpose of the robotic experiment is to be able to compare the usefulness of the human-in-the-loop reaction system^23^ with one relying on the robot’s low-level reaction controller, which uses machine learning without the human involvement when perceiving external stimuli. As this EEG system seems to offer a lower latency and could even anticipate very intense stimuli, our next step would be to connect the EEG part to the robotic approach by investigating the use of the developed brain features in real-time with the robotic prosthesis’ reaction system using the high sensitive robot skin, and validate the withdrawal reaction system when interacting in real-time with an embodied prosthesis^23^. Nonetheless, we are aware that one of the limitations of this study is the few subjects who participated in the experiment. Hence, generalizing the main finding of this work, including all comprehensive analyses, the identified patterns, as well as the high classification accuracy of the five different stimuli still needs to be further investigated and solidified. Nevertheless, we overcome this limitation by showing that most of our findings concur with the literature and previous studies whose purposes, goals, and used imaging techniques were different. Overall, this study provides initial significant findings supporting the efficacy of our paradigm.

## Methods

### Subject recruitment and sensory stimulation

**Figure 9:**
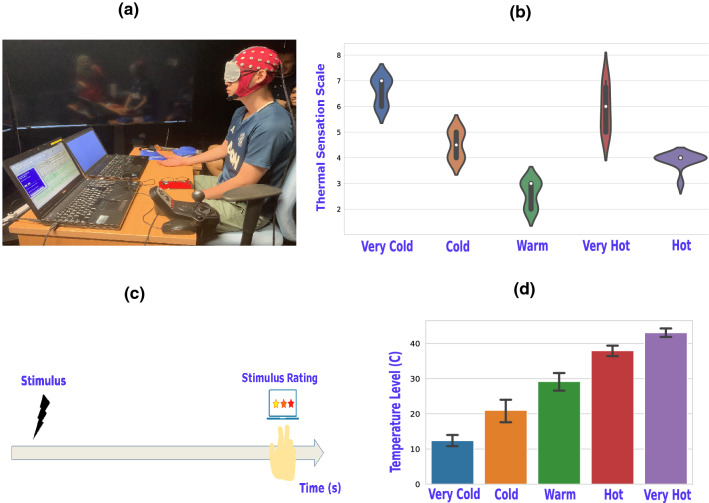
Experimental paradigm and data recording. (**a**) An example of a recording session of EEG during the thermal stimulation task. (**b**) A violin-plot presenting the average thermal sensation scoring of the five thermal stimuli across all the three subjects. (**c**) Experimental recording session starting with stimulus and concluded by a thermal sensation rating task during the recovery inter-trial break period. (**d**) A bar plot describing the mean and the standard deviation of the used temperature interval for each of the five thermal stimuli.

EEG data were recorded from three healthy volunteers (males) aged ${29.66 \pm 10.27}$ ${mean \pm SD, range [18--43] years)}$. All five thermal stimuli were delivered using customized thermal stimulators. It is worth noting that a sixth stimulation condition was recorded, where the three subjects were exposed to both NOX stimuli simultaneously. The results of this condition were investigated and presented separately in the Supplementary Information. Participants were asked to score the stimulus intensity on a well-validated thermal sensation scale-the visual analog scale^67^ ranging from 0 to 9 (9 being least tolerable). The average thermal sensation scoring across all subjects for the five thermal stimuli is depicted in Fig. 9b. All participants were right-handed, and hence all sensory stimulations were performed on their right arms. Temperature range for each stimulation condition is as follows: $[10\sim 14.99]$, $[16 \sim 24.99]$, $[25 \sim 33]$, $[35 \sim 40]$, $[40.99 \sim 44.99] ^5\;{^{\circ }}\hbox {C}$, for very cold^68^, cold^69^, warm (room temperature in Singapore), hot^70^, and very hot^70^, respectively, as illustrated in Fig. 9d.

### Research governance

This study was carried out in accordance with the Declaration of Helsinki. All experimental protocols were approved by the Institutional Review Board of the National Health Group, Singapore. All subjects were informed orally and in writing about the aims of the study, and a written consent for participation was sought and documented.

### EEG data recording and experiment

The study of brain activity evoked changes by five thermal stimuli was investigated using 64-channel EEG recorded data. To make sure that the subject was not substituting/anticipating the stimuli by sight, all subjects were blindfolded during the recordings. All recording sessions took place at the lab and were set up as shown in Fig. 9A. For each subject, two recording sessions were performed on two different days when each of the two NOX stimuli (very cold and very hot) were delivered on separate days. In the first recording session (day 1), very hot, hot, and warm stimuli were delivered for each subject (10 trials for each condition) yielding a total number of 30 trials. In the second recording session (day 2), very cold and cold stimuli were delivered for each subject, yielding 20 trials (10 trials for each condition). At the end of the second session of day 2 and in a separate investigation, each subject was exposed to both stimuli (very cold and very hot) simultaneously with a total number of 10 trials. Overall, 60 trials were recorded from all conditions, 50 trials for NOX, MOD, and INNO stimulations were processed and analyzed, whereas the remaining 10 trials for both hands stimulation conditions were analyzed separately and whose obtained results are presented in the Supplementary Information. Each trial consisted of three stages: baseline, stimulation, and recovery. It had a total duration of 40 s (10 s baseline and 30 s stimulation) where subjects were exposed to one of the five thermal stimulations separated by 2-min recovery breaks. Before each run, the surface temperature of the subject’s hand, as well as the thermal stimulator, were measured using an infrared thermometer. On each day, the total experiment lasted approximately 1.5 h. A Python script was used to display an automated visual cue informing the experimenter which thermal stimulus must be delivered. Each time, cues were pseudorandomized and were chosen from a discrete uniform distribution. An auditory warning beep preceded the cue display and was delivered five seconds before the commencement of the trial. Participants were asked to score the stimulus intensity during the recovery break at a scale from 0 to 9, as shown in Fig. 9c. During the experiment, participants were seated in a comfortable chair in a silent, temperature-controlled room and were instructed to not execute any movements. EEG data were collected using a 64 channel EEG device (Neuroscan system) with a 512 Hz sampling rate. The montage was in accordance with the 5% 10/20 system. Electrode impedance was kept below 10 kOhm in at least 95% of derivations throughout the experiment. The experimental paradigm is made publicly available with the gumpy software toolbox^71^. An animation video demonstrating the whole experiment and the main obtained results is available in the Supplementary Information.

### EEG signal processing and classification

The reference electrode was chosen on the vertex and the ground electrode was located on the forehead. Data were processed with custom designed Jupyter notebooks in Python using both gumpy^71^ and MNE^24,72^ toolboxes. For data analysis, 60 trials in total for the six stimulation conditions were considered. It should be noted that only 50 trials were used as the 10 trials that correspond to the both hands stimulation (very cold and very hot simultaneously) were analyzed separately and their results were presented in the Supplementary Information. EEG signals were band-pass-filtered between 0.5 and 70 Hz using a fourth-order Butterworth filter and notch filtered thereafter at 50 Hz. The cutoff frequencies Wc = (low = 0.0019, high = 0.273) were expressed as the fraction of half the sampling frequency (the Nyquist frequency) and the band corner frequencies. All signals were extracted from the recordings in 3000 ms epochs and used for further analysis. Epochs were baseline-corrected to the pre-stimulus mean^38^. Muscle artifacts were rejected by the Automatic Artifact Rejection (AAR)^73^ and independent component analysis (ICA) was used to remove eye movement artifacts^74^. Additionally, epochs containing high-amplitude artifacts or high-frequency muscle noise (visually inspected) were rejected from the analysis using a threshold-based method^75^. For that, EEG data beyond $100 \upmu V$ were removed, whereas recognized EMG artifacts (characterized by a high frequency component ranging from 50 to 200 Hz and high amplitude between 1 and 10 mV^76^) were manually eliminated. All EEG scalp topographies were plotted using the MNE toolbox, by matching channel location with its value given the defined latency. Topographies are color coded, where the green or yellow present null values, blue color presents negative values, and red encodes positive values; with the color intensity correlating with the channel value. Chosen time latencies in the topographic maps were based on an algorithm^24^ that computes and finds the highest peaks at each time point from all electrodes. For feature extraction and classifying the five conditions clustered in different binary groups from EEG, we implemented and tested a wide range of classical machine-learning approaches that are based on hand-crafted features. The common spatial patterns (CSP)^77^ was used as a feature extraction method. Linear Discrimination Analysis (LDA) was selected to classify the extracted features from EEG data. A PCA with only two components was used for dimensionality reduction, and the two components were fed thereafter to the different classifiers resulting in the presented 2D feature space (Fig. 5). During the test phase, data were divided into 80% for training and 20% for testing. As the total number of 50 trials was small when splitting the data into validation and test sets (only a few trials remained for each condition during the test phase), a data augmentation was performed^39^ for the classification task. The data augmentation formula is shown in equation 2. This yielded 10 separate trials from each one of the initial 50 trials (by using the whole 30 s and splitting them into a 3-s trial length), thus forming a total number of 500 trials. The 3-s trial length does not include the data before stimulation and the 3-s pre-stimulus phase were extracted separately from the baseline. It should be mentioned that the 500 trials (after data augmentation) were only used when performing the PCA, splitting the data, computing the test accuracy, as well as when doing the spectral analysis. Overall, 10-fold cross-validation was performed on training data to validate the model (validation accuracy) and the remaining 20% were used for the test phase. It should be noted for all analyses, bACC (shown in Eq. 4) was chosen as an evaluation metric for the trained models. bACC is calculated as the average of the proportion corrects of each class individually, where the same number of examples in each class was used.

### Source localization

MNE toolbox^24^ combined with gumpy^71^ Python toolbox were used for EEG processing and for source localization. First, cortical surface reconstruction using FreeSurfer^80^. Second, the forward solution and the forward model were computed using the boundary-element model (BEM)^81^. Thereafter, the regularized noise-covariance matrix, which gives information about potential patterns describing uninteresting noise source, was computed and estimated. Afterward, we computed the singular value decomposition (SVD) of the matrix composed of both estimated noise-covariance and the source covariance matrix. Finally, dSPM^32^ was computed and used for source localization and reconstruction. For dSPM, an anatomical linear estimation approach is applied. This assumes the sources are distributed in the cerebral cortex^82^. A linear collocation single-layer boundary-element method (BEM)^83^ is used to compute the forward solution which models the generated signal pattern at each location of the cortical surface. A noise-normalized minimum norm estimate is estimated at each cortical location resulting in an F-distributed estimation of the cortical current. Overall, dSPM identifies the locations of statistically increased current-dipolar strength relative to the noise level.

### Robotic experiment

#### Temperature level classification

Here, we develop a temperature level classifier capable of using the measured thermal data to predict the temperature range of any object in contact with the human-like skin^84^. For that, a robot skin patch^84^ consisting of two HEX-O-SKIN modules was used throughout the experiments as well as in the data gathering process. During the collection, the update rate of the robot skin patch was set to 250 Hz. In order to record temperature profiles, one of the five temperature levels was each time chosen randomly from a uniform distribution. Five everyday objects, each at a different temperature, were chosen for the experiment. All recording sessions were conducted at a room temperature of around $28\;^{\circ }\hbox {C}$ with all windows closed in order to keep the ambient temperature as consistent as possible throughout the recording sessions. For feature extraction and classifying the five conditions from EEG, we implemented and tested a wide range of classical machine learning approaches that are based on hand-crafted features. Five different classifiers from the gumpy.classification module^71^ were used and evaluated: K-Nearest Neighbor (KNN), Support vector machine (SVM), Naive Bayes (NB), LDA and Quadratic Linear Discrimination Analysis (QLDA). Five different feature extraction methods were initially investigated, namely mean absolute value (MAV), root mean square (RMS), variance (VAR), simple square integral, slope sign change, waveform length (WL), and Willison amplitude (WA). A feature selection algorithm^85^ was performed to select the most discriminating subset of features. The best features chosen for this classification task were MAV (formula is shown in 5) and VAR (formula is shown in 6) and the best classifier in terms of performance was QLDA. Data were split into 80% for training and 20% for the test phase. The classifier requires 3 s of contact between the robot skin and an object to predict one of the 5 different temperature classes based on the sensor measured data from this contact.

### Data analysis and statistics

All relevant information related to the obtained results is presented alongside their corresponding figures. For all statistical analyses, a normality check was first performed as well as data independence before choosing the adequate statistical test. The data normality (for the five thermal stimulation conditions) was checked using one-sample Kolmogorov–Smirnov test, which is a strict normality test, and as was suggested in the study by Strauss et.al^86^. Afterwards, the independence was checked using the Mann–Whitney U Test was used (frequently used when data is not normally distributed) and we found that the conditions are statistically independent (p $<0.001$). As two conditions were not normally distributed, we applied a Kruskal–Wallis test instead of ANOVA^86^. The Kruskal–Wallis test was combined with the post-hoc test for multiple group (pairwise) comparison. The software for the statistical analysis was implemented in python using the scipy and stats libraries. The Kruskal–Wallis H statistic was assumed to have a chi square distribution. No Tukey–Kramer correction for multi-group comparison was applied. Details are available in https://github.com/maximtrp/scikit-posthocs and https://scipy.stats.kruskal.html. For all the obtained results, we considered p $<0.05$ statistically significant to reject the null hypothesis. To study the correlation between alpha power decrease and stimulus intensity, the Pearson’s correlation coefficient (r) was applied (the formula is shown in Eq. 3). For that, the pearsonr() SciPy python function was used. The coefficient returns a value between − 1 and 1 that represents the limits of correlation from a full negative correlation to a full positive correlation^87^.

### Formulas and equations

#### GFP computation

The computed GFP shown in Fig. 3 is the standard deviation of the potentials at all EEG channels of an average given reference map^78^. The GFP formula is shown below in Eq. (1):

1 $$\begin{aligned} {\textit{GFP}} =\frac{\sqrt{ \sum \nolimits _{i=0}^\mathrm {N} ({\upmu }^{i}-{\overline{\upmu }}^2)}}{N}, \end{aligned}$$

where $\mu ^{i}$ is the voltage of the map $\mu$ for a given electrode i, ${\overline{\upmu }}$ is the mean voltage of all EEG electrodes of the map u and N is the number of electrodes of the map $\mu$. High GFP is explained by peak EEG activities as well as steep gradients. In Fig. 3, GFP shows the amount of activity at each time point in the field considering the data from all the 64 recording electrodes simultaneously^79^.

#### Data augmentation formula

For data augmentation, crops were created using a sliding window with a fixed length of, given the sampling frequency of 512 Hz. The sliding window shifts by *n* timesteps to create next crop until the end of the trial. Formally, given an original trial $\varvec{X}^j \in \mathbb {R^{E\cdot T}}$ with *E* electrodes and *T* timesteps, the sliding window generates crops $\varvec{C}^j$ with size $T'$ as slices of the original trial as follows:

2 $$\begin{aligned} \varvec{C}^j = \left\{ \varvec{X}^j_{[1,E], [t,t+T']} \vert t \in [1,T-T'] \right\} , \end{aligned}$$

where *j* is the trial index.

#### Pearson’s correlation coefficient (r) formula

The Pearson’s correlation coefficient (r) formula is shown below in equation 3:

3 $$\begin{aligned} r = \frac{ \sum _{i=1}^{n}(x_i-\bar{x})(y_i-\bar{y}) }{ \sqrt{\sum _{i=1}^{n}(x_i-\bar{x})^2}\sqrt{\sum _{i=1}^{n}(y_i-\bar{y})^2}}, \end{aligned}$$

where: *n* is sample size, $x_i$,$y_i$ are the individual sample points indexed with *i*, and $\bar{x}=\frac{\sum _{i=1}^{n}x_i}{n}$.

#### Balanced accuracy (bACC) formula

4 $$\begin{aligned} {\textit{bACC}} =\frac{Sensitivity + Specificity}{2}, \end{aligned}$$

where *Sensitivity* = True Positive/(True Positive + False Negative) and *Specificity* = True Negative/(True Negative + False Positive).

#### Statistical features formulas

Mean absolute value (MAV) formula:

5 $$\begin{aligned} MAV = \dfrac{1}{N} \sum \limits _{i=1}^{N} \vert x_{i} \vert . \end{aligned}$$

Variance (VAR) formula:

6 $$\begin{aligned} VAR = \dfrac{1}{N} \sum \limits _{i=1}^{N}\left( x_{i}-\overline{x}\right) ^{2}. \end{aligned}$$

## Supplementary Information

Supplementary Video 1.

Supplementary Video 2.

Supplementary Information.

## Data Availability

**Accession codes** All software codes will be made publicly available at https://github.com/gumpy-bci/gumpy.
