# Supplementary material for: Distinct spatio-temporal and spectral brain patterns for different thermal stimuli perception
Source: Sci Rep. 2022 Jan 18;12:919. doi: 10.1038/s41598-022-04831-w (PMC8766611; doi:10.1038/s41598-022-04831-w)
Supplement: Supplementary file 3 — Supplementary Information. [file 41598_2022_4831_MOESM3_ESM.pdf]

Distinct Spatio-Temporal & Spectral Brain Patterns for Different Thermal Stimuli Perception

Zied Tayeb[1,*], Andrei Dragomir[2, 3], Jin Ho Lee[1], Nida Itrat Abbasi[2], Emmanuel Dean[1, 4], Aishwarya Bandla[2], Rohit Bose[5], Raghav Sundar[2, 6], Anastasios Bezerianos[2, 7], Nitish V. Thakor[8, 9], and Gordon Cheng[1]

[1]Institute for Cognitive Systems, Technical University of Munich, Arcisstraße 21, 80333 München, Germany
[2]The N.1 Institute for Health, National University of Singapore, 28 Medical Dr. 05-COR, Singapore 117456
[3]Department of Biomedical Engineering, University of Houston, 3517 Cullen Blvd, Houston, TX 77204, USA
[4]Chalmers University of Technology- SE-412 96 Gothenburg, Sweden
[5]Department of Bioengineering, University of Pittsburgh, 3700 O'Hara Street, Pittsburgh, PA 15261, USA
[6]Department of Haematology-Oncology, National University Cancer Institute, National University Hospital, Singapore, 5 Lower Kent Ridge Rd, Singapore 119074
[7]Hellenic Institute of Transport (HIT), Centre for Research and Technology (CERTH), Thessaloniki, Greece
[8]Department of Biomedical Engineering, Johns Hopkins School of Medicine, 720 Rutland Ave, Baltimore, MD 21205, USA
[9]Department of Biomedical Engineering, National University of Singapore, Engineering Drive 3, 04-08, Singapore 117583
[*]zied.tayeb@tum.de

Power analysis of theta, beta, and gamma EEG frequency bands

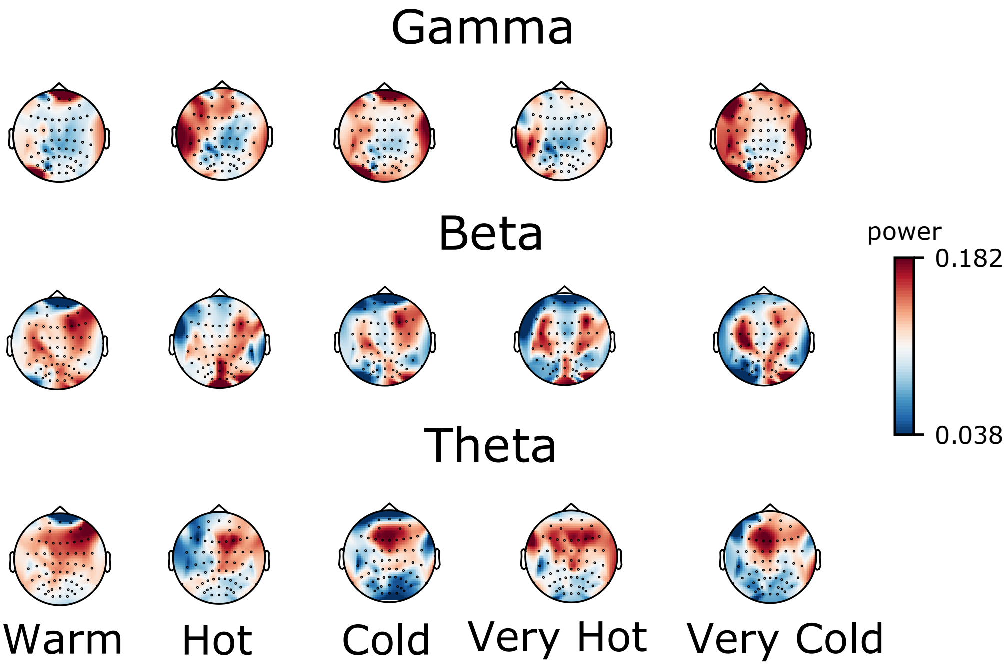

Figure 1. Obtained power results from theta, beta and gamma frequency bands.

Spatial-temporal patterns for the pre-stimulus phase

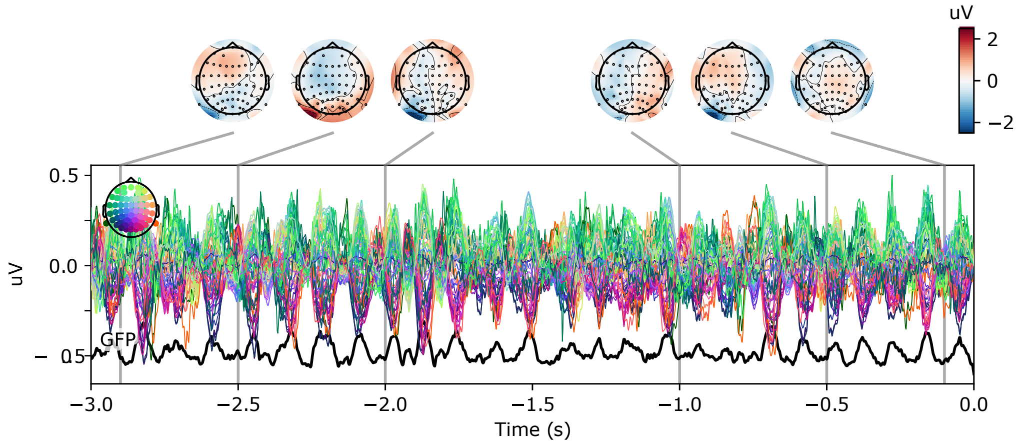

Figure 2. Spatial-temporal patterns for the pre-stimulus phase.

Very cold stimuli dominating very hot one when stimulating both hands

In a separate trial, the three subjects were exposed to both NOX stimuli at the same time. Very cold stimulus was delivered on the left hand, whereas very hot was delivered on the right hand. The very cold stimulus was scored more intense (scored 7 in average at a thermal sensation scale from 0 to 9) than the very hot stimulus (rated 4 in average using the same scoring scale). Interestingly, as illustrated in Figure 3 (A), the very cold showed higher contralateral decrease in alpha power (right side) than the very hot (left side). Similarly, higher frontal and central activity were detected for very cold (contralateral side to to the stimulation) compared to the very hot. These results confirm the main findings of the study, where a pronounced decrease of alpha power for both conditions but the most intense condition dominated the slightly intense one. This decrease was interpreted by an ERD resulting from a motor movement imagination. The high fronto-central activation for both conditions is in accordance with our main findings, revealing, therefore, the importance of the the regions in noxious stimuli thermal perception and processing. Investigations of the brain reaction when stimulating both hands with two different NOX stimuli should be further considered in future studies.

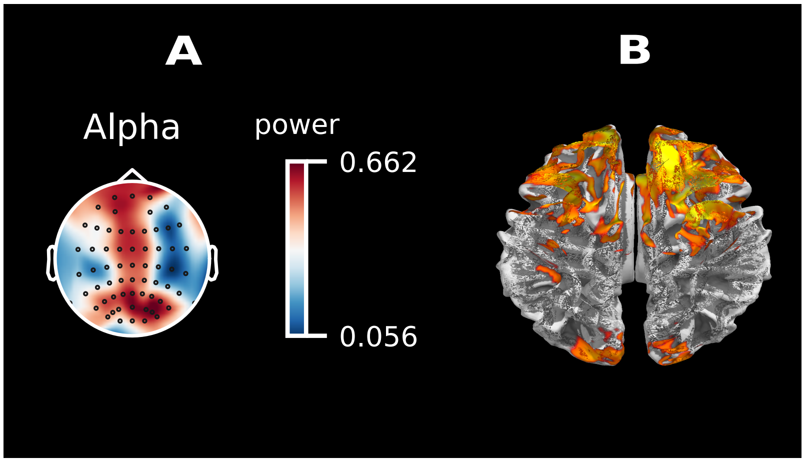

Figure 3. Both hands stimulation main results. **A**. EEG alpha power topographic map. **B**. EEG source localization analysis for all the three subjects.

Both stimuli thermal sensation scoring

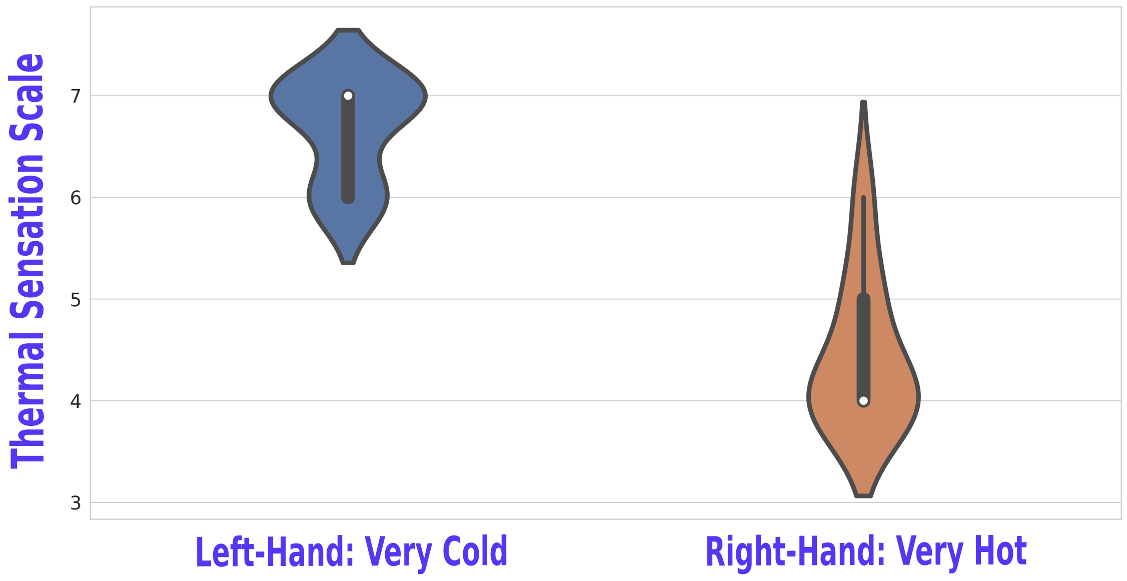

Figure 4. Both hand stimulations thermal sensation scoring from 0 to 9, where 9 is intolerable sensation.

Very Cold stimulation localized source for for the interval 200 to 500 ms post-stimulation

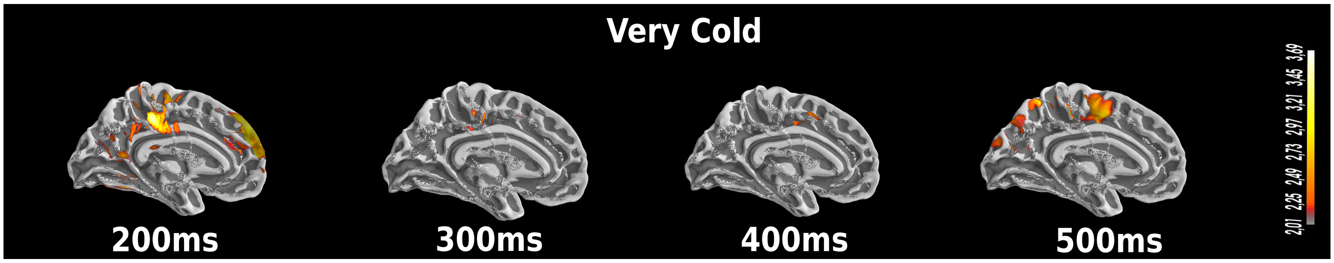

Figure 5. The most intense condition source localization for the time window [200 - 500] ms.
